# Supplementary material for: Acceptance and compliance with micronutrient powder (MNP) among children aged 6–23 months in northern Nigeria
Source: PLOS Glob Public Health. 2022 Oct 17;2(10):e0000961. doi: 10.1371/journal.pgph.0000961 (PMC10022258; doi:10.1371/journal.pgph.0000961)
Supplement: S1 Dataset — (ZIP) [file pgph.0000961.s008.zip › S8_Dataset/Spot Checks.zip!/Spot Checks/010715_A_SO_BA_2_SC.pdf]

SPOT CHECK RECORDING FORM

State: _ADAMAWA____ LGA: __SONG_________ Community: ___BOLKI____________

Name of caregiver (male)_____Mallam Ahmadu___________________________________

Sex of index child: ___FEMALE__ Age of index child (in months): ____6 MONTHS___

Spot Check Information

1. **Date of MNP distribution to household** (circle option below based on LGA name above)**:**
   - **Kebbi:** BirninKebbi&Jega LGA: **June 16**
   - **Kebbi:** Danko-wasagu: **June 17**
   - **Adamawa:**Song, Ganye: **June 18**
   - **Adamawa:**Guyuk **June 19**
2. **Date of Spot Check Observation: _01/07/2015__________________**
3. **Number of sachets remaining**(Ask the caregiver to show you and count the specific number of sachets remaining)**:**

   45

4. **Number of sachets that should remainusing one MNP sachet per day** (Analyst only)**:**

   48

5. **Number of sachets remaining / number of sachets should be remaining**(Analyst only)**:**

______45____ / ____48_____ = _____93.8____ %

Probes

Question 1. You have **XX** MNP sachets remaining. Explain how easy or difficult it was to use the MNP product on a daily basis for your child 6 – 23 months? (Circle one answer)

Very Difficult **Difficult** **Easy** **Very Easy**

Question 2. Describe any specific challenges that you faced that made the MNP difficult to use on a daily basis?

Question 3. Describe specific aspects of the MNP product that you or your child enjoyed that made it easier to use on a daily basis?

The MNP does not have any bitter taste that will make the child reject food containing it. It increased the child feeding behavior and she has added weight since she started taking the MNP.

Question 4. What questions do you have about the MNP based on your experience using it?

The man replied that the child has improved so far from my observation. He asked the wife who is preparing breakfast at the background for any side effect and she replied us that the only negative effect so far was diarrhea which was much when she started using the MNP and its subsiding gradually.
I replied them that it's the usual side effect experienced by some children whenever they start taking the MNP and there is no need for alarm since the diarrhea will stop on its own withing a few days.

Question 5. What specific recommendations do you have that we can do as a program to make the MNP easier to use on a daily basis?

The MNP should be prepared as a food that can be given to the child directly without having to mix it with food. Though the mixing with food isn't bad but I'm talking of a much better was to make it faster and easier to use. General acceptance so far isn't bad which means the procedure is convenient and user friendly.
